# Highly Efficient Catalysis of Azo Dyes Using Recyclable Silver Nanoparticles Immobilized on Tannic Acid-Grafted Eggshell Membrane

**DOI:** 10.1186/s11671-016-1647-7

**Published:** 2016-10-01

**Authors:** Xiaojing Liu, Miao Liang, Mingyue Liu, Rongxin Su, Mengfan Wang, Wei Qi, Zhimin He

**Affiliations:** 1 State Key Laboratory of Chemical Engineering, School of Chemical Engineering and Technology, Tianjin University, Tianjin, 300072 China; 2 Collaborative Innovation Center of Chemical Science and Engineering (Tianjin), Tianjin, 300072 China; 3 Tianjin Key Laboratory of Membrane Science and Desalination Technology, Tianjin, 300072 China

**Keywords:** Catalysis, Azo dyes, Eggshell membrane, Tannic acid, Silver nanoparticles

## Abstract

In this study, a facile one-step synthesis of a novel nanocomposite catalytic film was developed based on silver nanoparticles (AgNPs) immobilized in tannic acid-modified eggshell membrane (Tan-ESM). Tannic acid, as a typical plant polyphenol from oak wood, was first grafted onto ESM fibers to serve as both the reductant and the stabilizer during the synthesis of AgNPs. The morphology, constitution, and thermal stability of the resulting AgNPs@Tan-ESM composites were fully characterized to explain the excellent catalytic efficiency of AgNPs@Tan-ESM composites. These composite catalysts were applied to the degradation of azo dyes which exhibited the high catalytic activity toward Congo red and methyl orange according to the kinetic curves. More importantly, they can be easily recovered and reused for many times because of their good stability.

**Electronic supplementary material:**

The online version of this article (doi:10.1186/s11671-016-1647-7) contains supplementary material, which is available to authorized users.

## Background

Today, azo dyes have been extensively applied in the textiles, plastics, foods, drugs, cosmetics, and electronics industries [1]. Due to the toxic nature and poor biodegradability, azo dyes usually give rise to serious environment problems and health hazards during their manufacturing and storage. Thus, more and more attentions have been paid on the elimination of azo dyes through physical-chemical methods, such as biological degradation [2, 3], enzymatic degradation [4], Fenton-like process [5, 6], photocatalysis [7], and adsorption tactics [8, 9]. Among these, the biological degradation using anaerobic bacteria is usually suppressed in the presence of oxygen. And enzymatic treatments usually work on a specific type of dyes due to their narrow substrate specificity. Physical methods, such as active carbon adsorption or membrane filtration, usually lead to sludge which increase the operation cost. Photocatalysis seems to be more environmental friendly and energy saving [10, 11], but the practical applications are still limited because their activity are confined in ultraviolet range and the quantum yield is not satisfactory [12, 13].

Over the past few decades, metal nanoparticles have attracted considerable attention owing to their potential applications as catalysts [14–18]. The large surface-to-volume ratio endows metal nanoparticles with high activity. However, the small size brings some inevitable problems in separation and recycling. In order to overcome these problems, many efforts have been carried out to immobilize metal nanoparticles onto solid supports, such as inorganic oxides [19–21], insoluble polymers [22], hydrogel [23], and cross-linked protein crystal [24]. However, the synthetic polymer usually needs a complicated preparation process, and the hydrogel is fragile to the intensive agitation during reaction.

Eggshell membrane (ESM) is a kind of double-layered membrane in the inner side of eggshells. With the worldwide increasing consumption of eggs in food industry, a huge amount of eggshells have been thrown away as waste. However, as the bioresource, ESM is made up of more than 80 % proteins and hence has various functional groups. Besides, the intertwined fibrous network is mechanically stable which makes ESM an ideal support material for the immobilization of nanoparticles.

In the previous work, we have found that the physical adsorption on natural ESM is not strong enough to maintain the activity of silver nanoparticles (AgNPs) during reaction and recycling processing [24]. Therefore, it prompts us to develop a method to immobilize AgNPs on modified ESM so as to enhance the stability. Tannic acid, a natural plant polyphenol from oak wood, is considered to be a “green” reagent in the synthesis and immobilizing of AgNPs because the silver ions can be reduced by the phenolic hydroxyls under a mild condition without any other reducing agents.

Therefore, in the present work, a facile synthesis method was developed to immobilize AgNPs by grafting tannic acid onto the eggshell membrane (Tan-ESM) and in situ reducing silver ions without additional reductant. By this way, a stable linkage between AgNPs and ESM can be constructed to form the AgNPs@Tan-ESM composites. The physical and chemical properties of AgNPs@Tan-ESM composites were fully characterized. Finally, Congo red (CR) and methyl orange (MO) were chosen as the model substrates to investigate the catalytic behaviors of AgNPs@Tan-ESM composites in the degradation of azo dyes.

## Methods

### Materials

Fresh eggshells were collected from local market. Tannic acid (>98 %) was purchased from Guangfu Fine Chemical Research Institution (Tianjin, China). Glutaraldehyde (50 wt.%), silver nitrate (AgNO3, >99 %), sodium borohydride (NaBH4, >98 %), CR (>97 %), and MO (96 %) were purchased from Aladdin Reagent Co. (Shanghai, China). The ultrapure water (>18 MΩ cm) that prepared from a three-stage Millipore Milli-Q Plus 185 purification water system (Millipore Corp, Bedford, USA) was used throughout all the experiments.

### Preparation of Tan-ESM

ESM was carefully torn off from the inner side of fresh eggshell, cleaned with deionized water to remove the residual albumen, and stored in deionized water at 4 °C. In a typical procedure, ESM was cut into small pieces (5 mm × 5 mm) and vacuum dried at room temperature. Then, 0.5 g of dried ESM pieces was dispersed into 50.0-mL deionized water containing a certain amount of tannic acid under the continuous stirring at 37 °C. After 2 h, 0.75-mL glutaraldehyde was added into the mixture; the reaction was kept stirring for 6 h at 37 °C until the color of ESM turned yellow. The obtained Tan-ESM pieces were separated and washed with deionized water for several times to remove the unreacted tannic acid. Finally, Tan-ESM was vacuum dried at room temperature and ready for the following experiments.

### Preparation of AgNPs@Tan-ESM Composites

The as-prepared dried Tan-ESM pieces were dispersed into 50.0 mL AgNO_3_ solution (10 mM) and stirred at 37 °C. After 12 h, the obtained AgNPs@Tan-ESM composites were separated, washed with deionized water, and vacuum dried.

### Degradation of Azo Dyes Catalyzed by AgNPs@Tan-ESM

In a typical catalytic experiment, 11 mg of AgNPs@Tan-ESM composites was dispersed into a freshly prepared solution containing 8 mL deionized water and 500 μL CR or MO solution (3 mM). N_2_ was piped into the solution for 10 min to remove the dissolved oxygen. After the preheating at 40 °C, 0.9 mL of NaBH_4_ solution (0.3 mM) was rapidly injected into this mixture to start the reaction. The reaction was monitored by UV-vis absorption spectra (Persee TU-1810, China) in the range of 250–700 nm through withdrawing samples from reaction mixture at each time interval.

### Characterization

The surface morphologies of natural ESM, Tan-ESM, and AgNPs@Tan-ESM composites were characterized by scanning electron microscopy (SEM; S-4800, Hitachi Ltd.) at an accelerating voltage of 3.0 kV equipped with an energy-dispersive X-ray (EDX). All samples were vacuum dried before determination. The morphology of the AgNPs was characterized by a high-resolution transmission electron microscopy (HRTEM, JEM-2100 F, 200 kV). A sufficient dispersed AgNP sample was obtained through high-intensive ultrasonic treatment of AgNPs@Tan-ESM composites in water. Then, a droplet of dispersions was dropped on a carbon-coated copper grid and dried at room temperature. The X-ray diffract (XRD) pattern were collected on an X-ray diffract meter (D/max 2500, Rigaku) with a Cu Kα radiation. The thermogravimetric analysis (TGA) of natural ESM, Tan-ESM, and AgNPs@Tan-ESM composites were carried out on a simultaneous TGA-DTA apparatus (PTC-10A, Rigaku, Japan) through gradually heating the samples from the room temperature to 700 °C at the rate of 10 °C/min. The attenuated total reflectance fast Fourier transformation infrared (ATR-FTIR, Nicolet Nexus 670) spectrum from 4000 to 400 cm^−1^ was collected to analyze the functional groups of the samples.

## Results and Discussion

### Preparation of AgNPs@Tan-ESM Composites

**Scheme 1:**
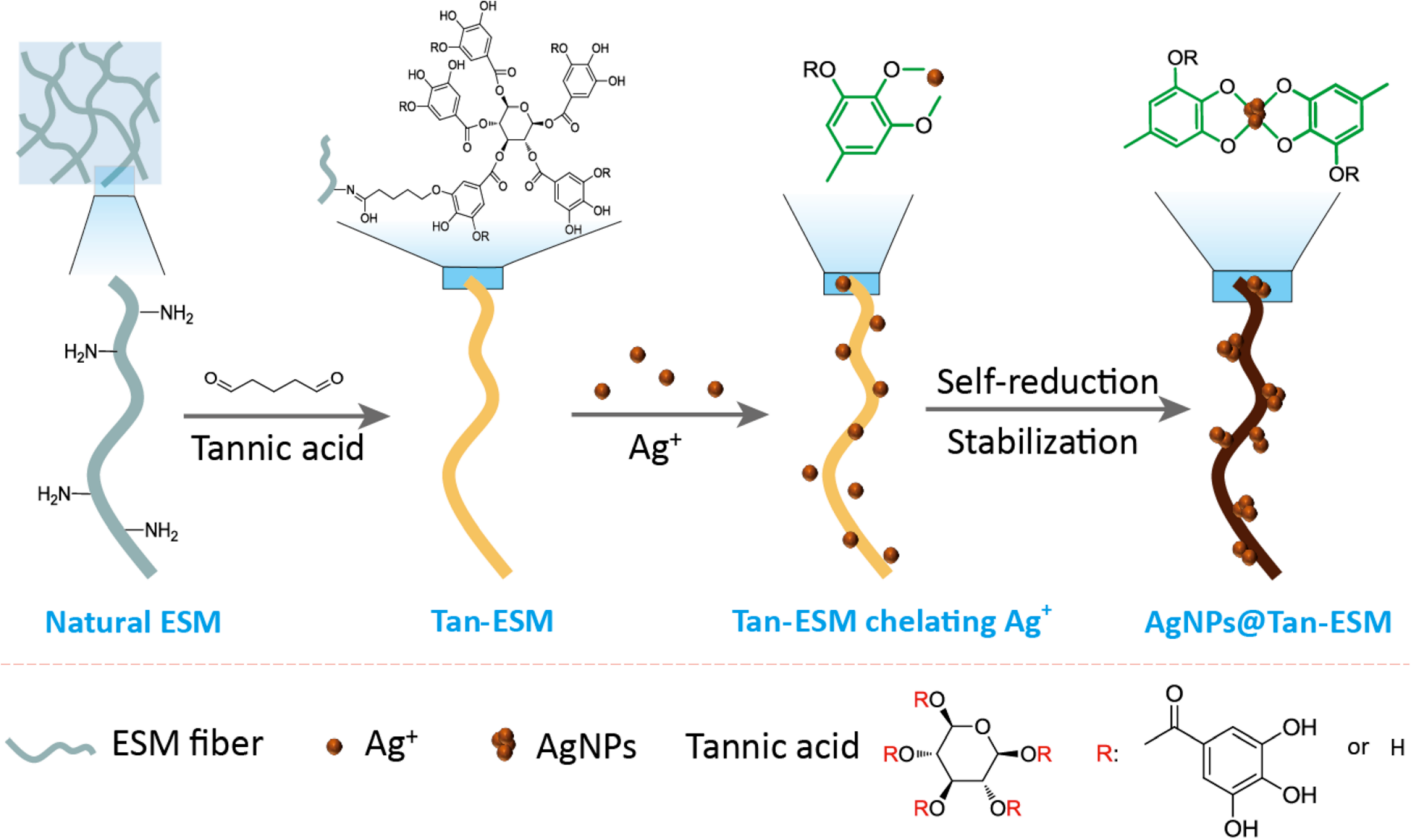
Schematic diagram for the preparation of AgNPs@Tan-ESM composites

The detailed synthesis process of AgNPs@Tan-ESM composites is shown in Scheme 1. In this approach, Tan-ESM was firstly obtained through grafting tannic acid onto the surface of ESM. By using glutaraldehyde as cross-linking agent, covalent bindings formed both between the aldehyde group on glutaraldehyde and the amino group on ESM as well as the aldehyde group on glutaraldehyde and the hydroxyl group on tannic acid. As a result, tannic acid was grafted on the surface of ESM leading to a color change of ESM from white to yellow.

### Characterization of AgNPs@Tan-ESM Composites

**Fig. 1:**
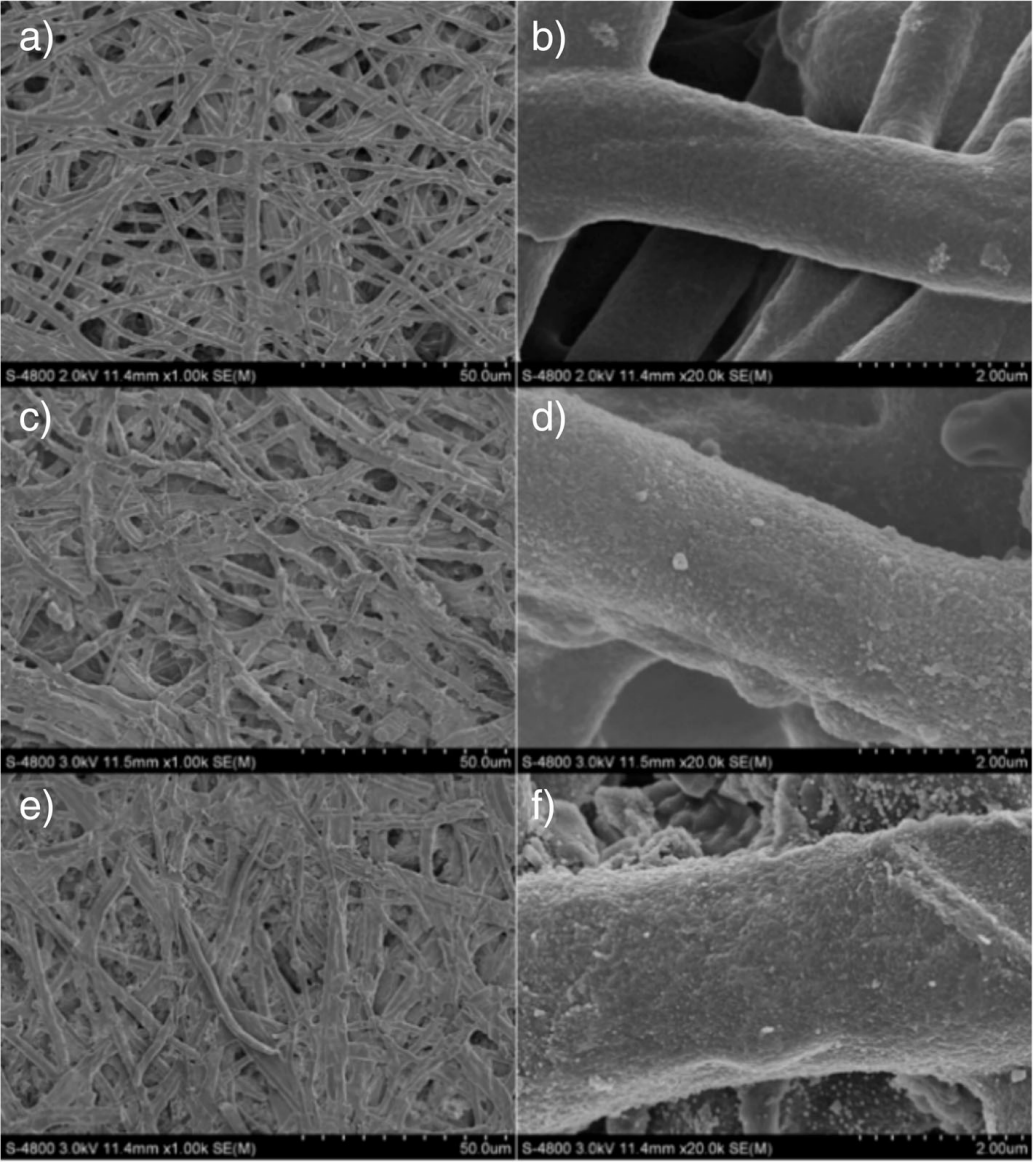
The SEM images of natural ESM (**a**, **b**), Tan-ESM (**c**, **d**), and AgNPs@Tan-ESM composites (**e**, **f**) at different magnifications

The SEM images show the morphological changes of ESM after grafting. As shown in Fig. 1a, b, the natural ESM presents the network structure which composed of interlaced protein fibers with the diameters of 0.5–2 μm and smooth surfaces. After the grafting of tannic acid, rough surface was observed on ESM from both the macroscopic hierarchical structure (Fig. 1c) and the highly magnified image (Fig. 1d). This phenomenon proved that tannic acid has been successfully linked onto ESM.

As a typical plant polyphenol, tannic acid is rich in orthophenolic hydroxyl groups which make it a preeminent ligand to chelate Ag ions and form the stable five-member chelating rings [25]. Compared with procyanidine that reported in our previous work [26], tannic acid provides more binding sites for metal nanoparticles. Due to the reducing capacity of orthophenolic hydroxyls in tannic acid, the Ag ions can be spontaneously reduced to Ag^0^ atoms without adding other reducing agents. With the in situ nucleation and growth, Ag^0^ formed AgNPs and could be stabilized through interacting with the oxygen atoms on tannic acid molecules. Therefore, the color of ESM was found turning from yellow to deep brown, confirming the formation of AgNPs@Tan-ESM composites.

The network structure of ESM provides a large specific surface area for AgNPs to nucleate and grow. As shown in Fig. 1e, f, the network structure was remained after the reduction reaction and lots of nanoparticles were found decorated on the fiber surface which make the fiber surface even rougher. Therefore, it is believed that ESM is a robust biomatrix for the immobilization of nanoparticles. The prominent reducibility of tannic acid and the excellent stability of ESM facilitate the fabrication of AgNPs@Tan-ESM composites.

**Fig. 2:**
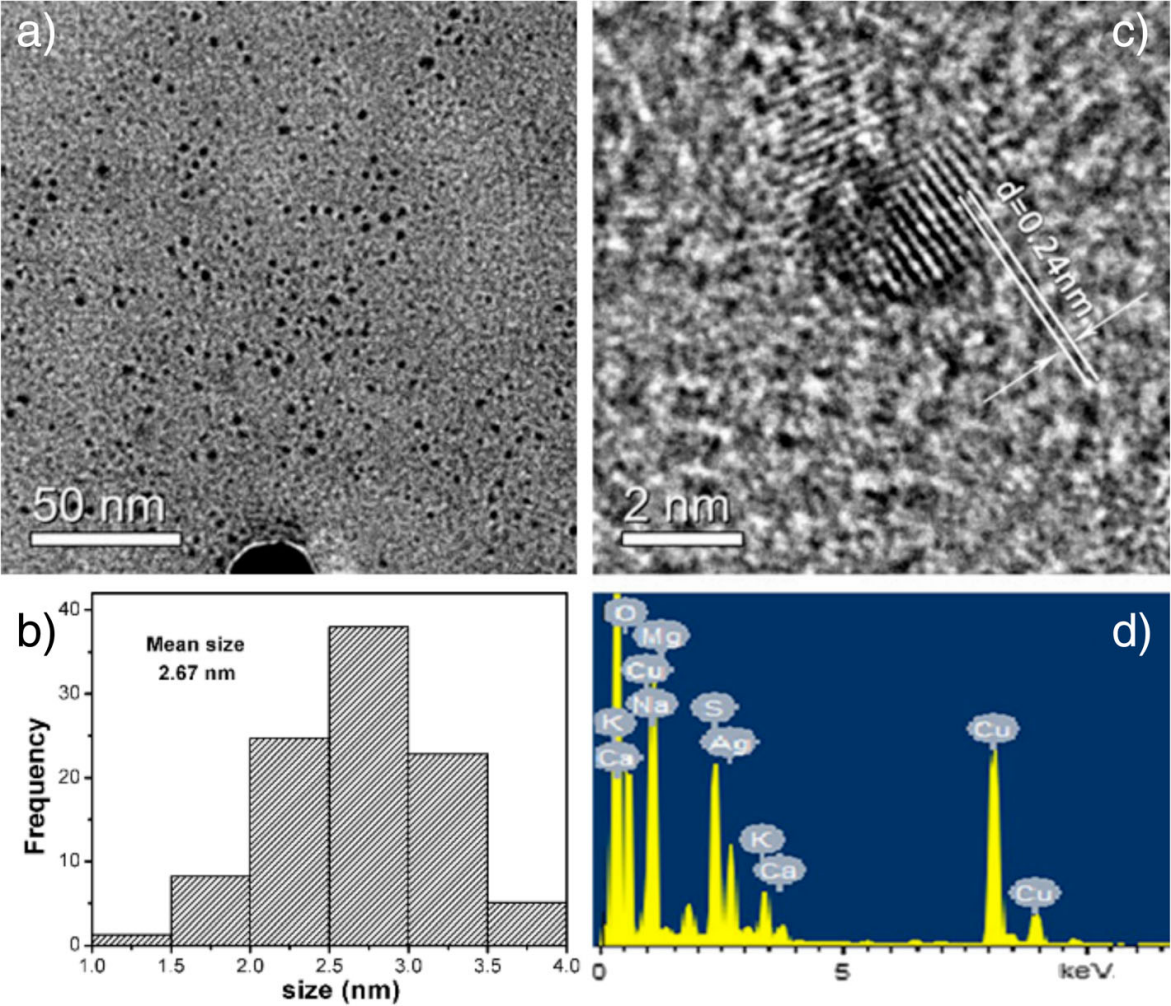
The SEM image of AgNPs (**a**), the size distribution of AgNPs (**b**), typical HRTEM image of AgNPs (**c**), and the EDX patterns of AgNPs@Tan-ESM composites (**d**)

In order to investigate the characteristics of nanoparticles in AgNPs@Tan-ESM composites, transmission electron microscopy (TEM) was used to determine the microstructure and the particle size of AgNPs. Since it is technically difficult to analyze nanoparticles directly on a certain thickness of ESM, the AgNPs were extracted from AgNPs@Tan-ESM composites through high-intensive ultrasonic treatment in water. The TEM image reveals that the shape of AgNPs is spherical and the size of them is approximately 1–4 nm (Fig. 2a). A statistical histogram (Fig. 2b) was obtained through analyzing the TEM image with ImageJ software. It is demonstrated that the AgNP has a narrow size distribution with a mean size of 2.67 nm. This small size leads to a huge surface area-to-volume radio which contributes to the high catalytic activity of AgNPs [27]. A typical HRTEM image of the individual particle (Fig. 2c) claims the crystalline structure of AgNPs with the lattice spacing of 0.24 nm. This corresponds to the (111) plane in face-centered cubic (fcc) structure which indicates the formation of crystalline AgNPs [24, 28]. Additionally, the elemental composition of AgNPs@Tan-ESM composites was detected by EDX (Fig. 2d) where the peak of Ag element was obviously observed, confirming the AgNPs were successfully imported into AgNPs@Tan-ESM composition.

**Fig. 3:**
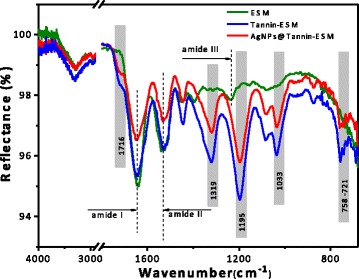
ATR-FTIR spectra of natural ESM (*green line*), Tan-ESM (*blue line*), and AgNPs@Tan-ESM composites (*red line*)

ATR-FTIR was used to analyze the functional groups of natural ESM, Tan-ESM, and AgNPs@Tan-ESM composites (Fig. 3). The absorption peaks at 1650, 1525, and 1232 correspond to the amide I (C=O stretching vibration), amide II (N–H in plane bending/C–N stretching vibration), and amide III, respectively [29]. This refers to the characteristic peaks of protein which comes from the natural eggshell membrane.

Comparing with natural ESM, several new peaks appeared in the spectra of Tan-ESM and AgNPs@Tan-ESM composites. The peaks at 1716 and 1319 cm^−1^ reflect the form of Ar–COOR, the peak at 1033 cm^−1^ corresponds to the C–O–C stretching vibration of the benzene ring, and the peak at 1195 cm^−1^ owes to the C–O–H stretching vibration of phenolic hydroxyls. Besides, the characteristic peaks of long-chain alkane at 758–721 cm^−1^ reflect the existence of glutaraldehyde. All these results indicated that the tannic acid has been successfully grafted on the surface of the ESM. Comparing with Tan-ESM, the transmittance of AgNPs@Tan-ESM composites was slightly depressed around 3300 cm^−1^, indicating the interactions between Tan-ESM and AgNPs.

**Fig. 4:**
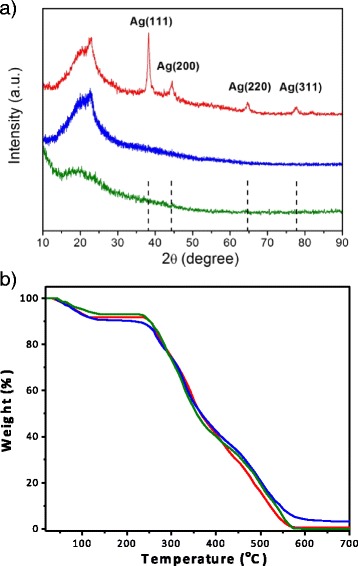
The XRD pattern (**a**) and the TGA curve (**b**) of natural ESM (*green line*), Tan-ESM (blue line), and AgNPs@Tan-ESM composites (*red line*)

XRD analysis was performed in order to further verify the existing of AgNPs on the surface of Tan-ESM. As Fig. 4a shows, a typical XRD pattern with specific diffraction peaks at 2*θ* = 38.1°, 44.3°, 64.5°, and 77.4° were obtained for AgNPs@Tan-ESM composites which attribute to the (1 1 1), (2 0 0), (2 2 0), and (3 1 1) reflections of cubic (fcc) structure. This demonstrates the crystalline AgNPs have already formed on the surface of Tan-ESM. Besides, the broad peak at 2*θ* = 21° was observed for all the samples, indicating the amorphous phase of protein fibers, which is consistent with the conformation and sequence of amino acids of proteins on ESM as reported previously [30].

Furthermore, TGA was performed to investigate the thermal stability of natural ESM, Tan-ESM, and AgNPs@Tan-ESM composites as shown in Fig. 4b. All the samples were gradually heated from room temperature to 700 °C at the rate of 10 °C/min. Due to the releasing of the bound water, the weight of all the samples decreased slowly before 250 °C. Beyond 250 °C, the weight began to drop sharply which demonstrated the drastic decompose of ESM until 570 °C. The identical TGA curves of ESM, Tan-ESM, and AgNPs@Tan-ESM composites imply their similar thermal stability. Based on the TGA curve, the AgNP content in AgNPs@Tan-ESM composite can be approximately calculated as 3.3 %. It is almost twice the content of AgNPs on AgNPs@ESM composites (1.64 %) which immobilized AgNPs on the plain ESM through physical absorption [24].

### Catalytic Degradation of Azo Dyes

Azo dyes are the most important synthetic colorants which have been widely used in textile, printing, paper manufacturing, etc. However, azo dyes can be easily broken down to aromatic amine, which leads to a serious risk to environment. In this study, CR and MO were chosen as the typical reaction substrate to evaluate the catalytic ability of AgNPs@Tan-ESM composites in the degradation of azo dyes.

**Fig. 5:**
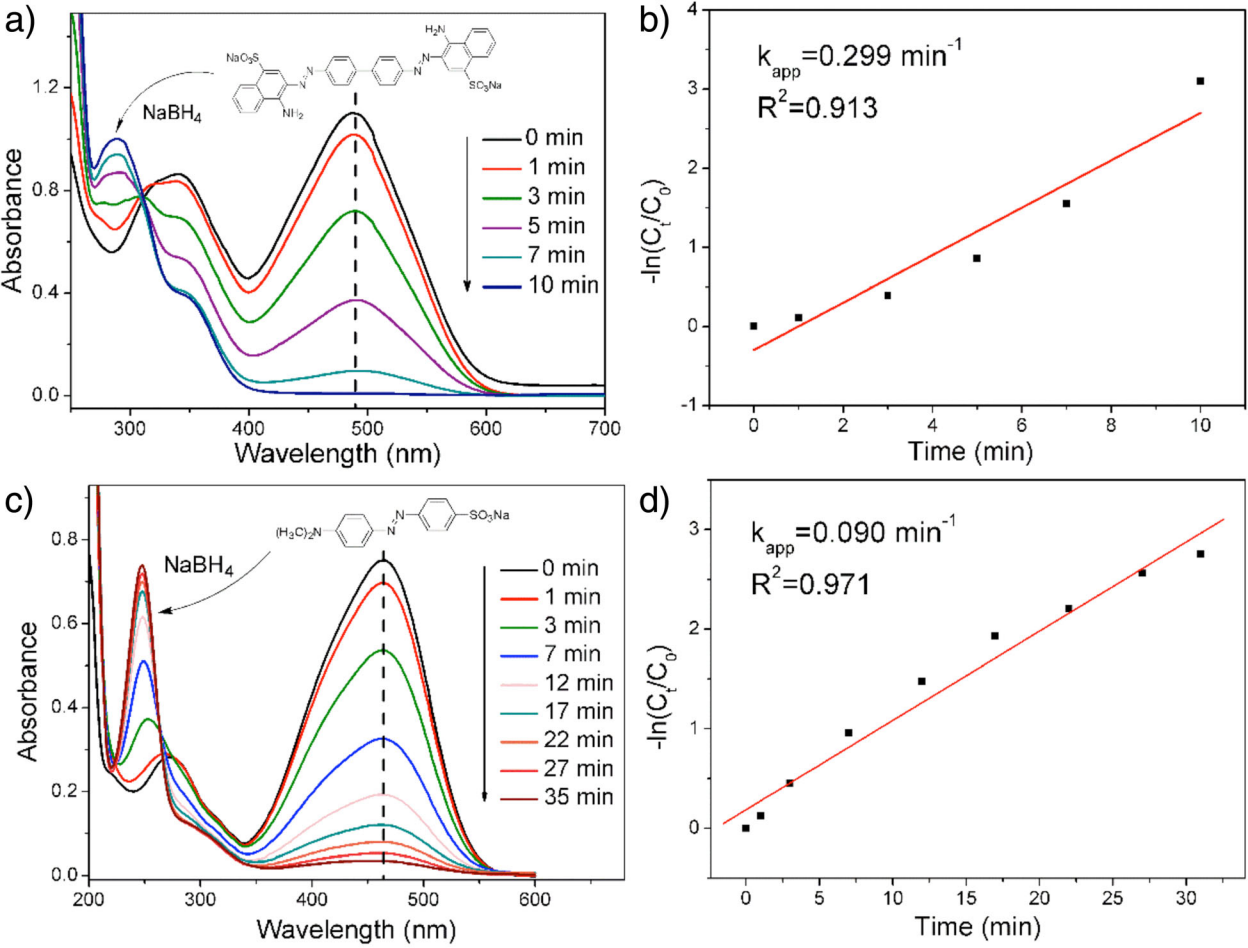
The time-dependent UV-vis absorption spectra of azo dyes degraded by AgNPs@Tan-ESM composites (**a**, **c**) and the kinetic curve (**b**, **d**) in the process of reaction (**a** and **b** refer to Congo red, **c** and **d** refer to methyl orange)

Figure 5a, c presents the typical time-dependent UV-vis absorption spectra during the reaction. It is found that the maximum absorption peak of CR (*λ*_max_ = 488 nm) and MO (*λ*_max_ = 446 nm) declined rapidly when using AgNPs@Tan-ESM composites as catalyst. At the meantime, the absorption peak near 290 nm (Fig. 5a) and 250 nm (Fig. 5c) increased, implying the CR and MO have already been converted into other substances. As a result, the reaction solutions became transparent after 10 and 35 min which indicated the azo dyes have already been eliminated by AgNPs@Tan-ESM composites. According to Additional file 1: Figure S1, the catalytic effects of AgNPs@Tan-ESM come from the AgNPs because the Tan-ESM does not show any effects on the reduction of CR or MO. As a catalyst, AgNPs accept the electrons from BH_4_^−^ and then transfer the electrons to azo dyes. Therefore, the reduction of azo dyes takes place only when both species (NaBH_4_ and azo dyes) are absorbed on the surface of the catalyst (AgNPs). As expected, there was no significant change in the UV-vis absorption spectra of CR and MO after adding only AgNPs@Tan-ESM or NaBH_4_ (Additional file 1: Figure S2). Owing to the network structure of ESM, the mass transfer limitation was suppressed which facilitate the binding of substrates and the release of products.

Given that NaBH_4_ significantly exceeds in the reaction, the concentration of NaBH_4_ is assumed to be constant and the rate of the reaction only depends on the concentration of azo dyes. The consumption of CR and MO can be given as:

1 $$ -{r}_{\mathrm{CR}}=\frac{-d{C}_{\mathrm{CR}}}{dt}={k}_{\mathrm{CR}}\cdot {C}_{\mathrm{CR}} $$

2 $$ -{r}_{\mathrm{MO}}=\frac{-d{C}_{\mathrm{MO}}}{dt}={k}_{\mathrm{MO}}\cdot {C}_{\mathrm{MO}} $$

where *r*_CR_ and *r*_MO_ are the consumption rates of CR and MO at a certain time of *t*, *C*_CR_ and *C*_MO_ are the concentrations of CR and MO at *t*, and *k*_CR_ and *k*_MO_ are the reaction rate constants.

Thus, the rate constants of the pseudo-first-order reaction can be obtained through simple integral:

3 $$ - ln\left({C}_{CR}/{C}_0\right)={k}_{CR}\times t $$

4 $$ - ln\left({C}_{MO}/{C}_0\right)={k}_{MO}\times t $$

Figure 5b, d shows the kinetic curves for the degradation of CR and MO. Linear relationship between ln(C_t_/C_0_) and reaction time suggested that the degradation reaction abides by the pseudo-first-order kinetics model, and the rate constants for CR (*k*_CR_ = 0.299 min^−1^) and MO (*k*_MO_ = 0.090 min^−1^) can be calculated as described in supporting materials. Comparing with the AgNPs immobilized on silica (*k*_MO_ = 0.035 min^−1^) [31], the synthesis AgNPs@Tan-ESM composites displayed excellent catalytic activity in the degradation of MO at a low molar ratio of NaBH_4_ to dyes. This is because the network structure of ESM contributes to the forming of small size AgNPs and the high loading of AgNPs.

### Catalyst Stability and Reuse

**Fig. 6:**
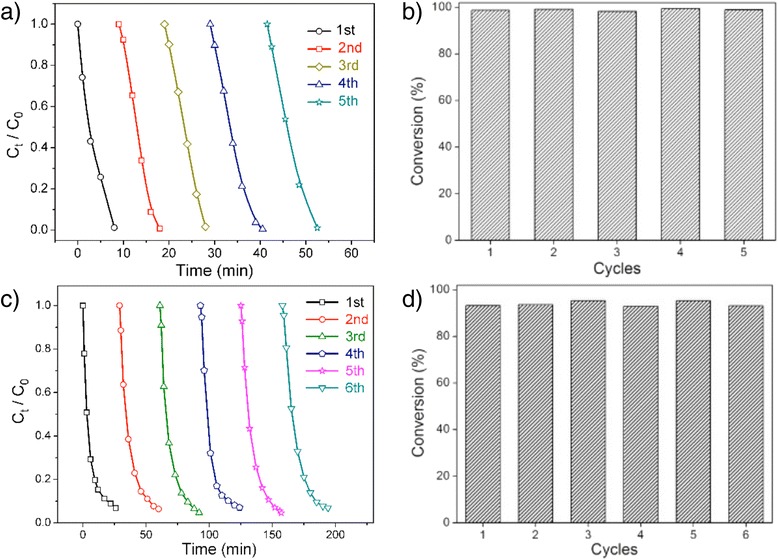
Catalytically recyclable degradation of azo dyes as a function of time (**a**, **c**) and the conversion ratios of azo dyes (**b**, **d**) in several successive cycles of reaction (**a** and **b** refer to Congo red, **c** and **d** refer to methyl orange)

Recyclability is an important property for catalysts in the industrial application. In the case of nanoscaled catalysts, agglomeration usually happens during the separation and recovery process which unfortunately suppresses the activity of nanoparticles. For AgNPs@Tan-ESM composites, the network structure of ESM provides a robust carrier to support the AgNPs which facilitates the separation of the catalysts from reaction system without any agglomeration. Besides, owing to the rich orthophenolic hydroxyl groups in tannic acid, Tan-ESM exhibits a strong chelating capacity to stabilize the nanoparticles and prevent them from leaking. Therefore, the AgNPs@Tan-ESM composites can be easily recovered through filtration and reused in the next reaction cycle. Figure 6a, b shows the recyclability of AgNPs@Tan-ESM composites in the degradation of CR. It is found that the reaction time was still less than 12 min and the conversion of CR retained 98 % by using AgNPs@Tan-ESM composites for at least 5 cycles. The similar result was obtained for the degradation of MO as shown in Fig.6c, d. During the recycling process, both the reaction rate and the conversion were kept at a high level after 6 cycles. These results clearly demonstrated that Tan-ESM is an excellent carrier to immobilized AgNPs. As shown in Fig. 6a, c, the time taken for the degradation of the dyes increases with the reused times, which may probably be attributed to the poisoning of the nanocrystal surface by adsorption of reactants or products [32].

## Conclusions

In this study, tannic acid was firstly grafted onto ESM so as to in situ reduce Ag ions into AgNPs and subsequently immobilize the formed AgNPs onto the surface of ESM. As a catalyst, the resulting AgNPs@Tan-ESM composites exhibited excellent catalytic activity for the degradation of azo dyes in aqueous phase. Moreover, due to the stability of ESM, AgNPs@Tan-ESM composites can be easily recovered and reused at a high catalytic efficiency. By combing the merits of tannic acid and ESM, a novel strategy was presented to obtain an environmental friendly, simple, and effective catalyst for the environmental treatment of azo dyes.

## Additional file

Additional file 1: Figure S1. The catalytic effects of Tan-ESM and AgNPs@Tannin-ESM composites on the reduction of CR (488 nm) and MO (446 nm). Figure S2. The time-dependent UV-vis absorption spectra of Congo red (a, c) and methyl orange (b, d) using AgNPs@Tan-ESM and NaBH_4_ separately. (DOCX 194 kb)

## References

[1] Buitrón G, Quezada M, Moreno G (2004). Aerobic degradation of the azo dye acid red 151 in a sequencing batch biofilter. Bioresour Technol.

[2] Forss J, Pinhassi J, Lindh M, Welander U (2013). Microbial diversity in a continuous system based on rice husks for biodegradation of the azo dyes Reactive Red 2 and Reactive Black 5. Bioresour Technol.

[3] Hai FI, Yamamoto K, Nakajima F, Fukushi K, Nghiem LD, Price WE, Jin B (2013). Degradation of azo dye acid orange 7 in a membrane bioreactor by pellets and attached growth of Coriolus versicolour. Bioresour Technol.

[4] Malani RS, Khanna S, Moholkar VS (2013). Sonoenzymatic decolourization of an azo dye employing immobilized horse radish peroxidase (HRP): a mechanistic study. J Hazard Mater.

[5] Bouasla C, Samar ME-H, Ismail F (2010). Degradation of methyl violet 6B dye by the Fenton process. Desalination.

[6] Yao Y, Wang L, Sun L, Zhu S, Huang Z, Mao Y, Lu W, Chen W (2013). Efficient removal of dyes using heterogeneous Fenton catalysts based on activated carbon fibers with enhanced activity. Chem Eng Sci.

[7] Zhang Y, Zhu J (2015). Composite photocatalytic membrane prepared by embedding porous SiO_2_ shell/void/TiO_2_ core particles into polycarbonate for photodegrading and removing pollutant from water. Chem Eng Sci.

[8] Iqbal MJ, Ashiq MN (2007). Adsorption of dyes from aqueous solutions on activated charcoal. J Hazard Mater.

[9] Özbay İ, Özdemir U, Özbay B, Veli S (2013). Kinetic, thermodynamic, and equilibrium studies for adsorption of azo reactive dye onto a novel waste adsorbent: charcoal ash. Desalin Water Treat.

[10] Rauf MA, Meetani MA, Hisaindee S (2011). An overview on the photocatalytic degradation of azo dyes in the presence of TiO_2_ doped with selective transition metals. Desalination.

[11] de Souza ML, Corio P (2013). Effect of silver nanoparticles on TiO_2_-mediated photodegradation of Alizarin Red S. Appl Catal B.

[12] Zhang J, Xiong Z, Zhao XS (2011). Graphene–metal–oxide composites for the degradation of dyes under visible light irradiation. J Mater Chem.

[13] Zhang Y, Chen Z, Liu S, Xu Y-J (2013). Size effect induced activity enhancement and anti-photocorrosion of reduced graphene oxide/ZnO composites for degradation of organic dyes and reduction of Cr(VI) in water. Appl Catal B.

[14] Bokare AD, Chikate RC, Rode CV, Paknikar KM (2008). Iron-nickel bimetallic nanoparticles for reductive degradation of azo dye Orange G in aqueous solution. Appl Catal B.

[15] Qian H, Jiang DE, Li G, Gayathri C, Das A, Gil RR, Jin R (2012). Monoplatinum doping of gold nanoclusters and catalytic application. J Am Chem Soc.

[16] Yola ML, Eren T, Atar N, Wang S (2014). Adsorptive and photocatalytic removal of reactive dyes by silver nanoparticle-colemanite ore waste. Chem Eng J.

[17] Gao Z, Su R, Huang R, Qi W, He Z (2014). Glucomannan-mediated facile synthesis of gold nanoparticles for catalytic reduction of 4-nitrophenol. Nanoscale Res Lett.

[18] Wu S, Yan S, Qi W, Huang R, Cui J, Su R, He Z (2015). Green synthesis of gold nanoparticles using aspartame and their catalytic activity for p-nitrophenol reduction. Nanoscale Res Lett.

[19] Samanta A, Dhar BB, Devi RN (2012). Ultrasmall gold cluster arrays encapsulated in silica nanospheres: applications in fluorescence imaging and catalysis. J Phys Chem C.

[20] Dong Z, Le X, Li X, Zhang W, Dong C, Ma J (2014). Silver nanoparticles immobilized on fibrous nano-silica as highly efficient and recyclable heterogeneous catalyst for reduction of 4-nitrophenol and 2-nitroaniline. Appl Catal B.

[21] Ji Z, Ismail MN, Callahan DM, Pandowo E, Cai Z, Goodrich TL, Ziemer KS, Warzywoda J, Sacco A (2011). The role of silver nanoparticles on silver modified titanosilicate ETS-10 in visible light photocatalysis. Appl Catal B.

[22] Hyun DC, Park M, Park C, Kim B, Xia Y, Hur JH, Kim JM, Park JJ, Jeong U (2011). Ordered zigzag stripes of polymer gel/metal nanoparticle composites for highly stretchable conductive electrodes. Adv Mater.

[23] Zheng Y, Wang A (2012). Ag nanoparticle-entrapped hydrogel as promising material for catalytic reduction of organic dyes. J Mater Chem.

[24] Liang M, Wang L, Su R, Qi W, Wang M, Yu Y, He Z (2013). Synthesis of silver nanoparticles within cross-linked lysozyme crystals as recyclable catalysts for 4-nitrophenol reduction. Catal Sci Technol.

[25] Huang X, Wu H, Liao X, Shi B (2010). One-step, size-controlled synthesis of gold nanoparticles at room temperature using plant tannin. Green Chem.

[26] Liang M, Su R, Huang R, Qi W, Yu Y, Wang L, He Z (2014). Facile in situ synthesis of silver nanoparticles on procyanidin-grafted eggshell membrane and their catalytic properties. ACS Appl Mater Interfaces.

[27] Wu H, Huang X, Gao M, Liao X, Shi B (2011). Polyphenol-grafted collagen fiber as reductant and stabilizer for one-step synthesis of size-controlled gold nanoparticles and their catalytic application to 4-nitrophenol reduction. Green Chem.

[28] Ji T, Chen L, Mu L, Yuan R, Knoblauch M, Bao FS, Zhu J (2016). In-situ reduction of Ag nanoparticles on oxygenated mesoporous carbon fabric: exceptional catalyst for nitroaromatics reduction. Appl Catal B.

[29] Guli M, Lambert EM, Li M, Mann S (2010). Template-directed synthesis of nanoplasmonic arrays by intracrystalline metalization of cross-linked lysozyme crystals. Angew Chem Int Ed.

[30] Su HL, Xu J, Chen JJ, Moon WJ, Zhang D (2012). In situ formation and assembly of CdS nanocrystallites into polyhedrons on eggshell membrane at room temperature. Appl Phys A Mater Sci Process.

[31] Badr Y, Mahmoud MA (2007). Photocatalytic degradation of methyl orange by gold silver nano-core/silica nano-shell. J Phys Chem Solids.

[32] Yu T, Zeng J, Lim B, Xia Y (2010). Aqueous-phase synthesis of Pt/CeO_2_ hybrid nanostructures and their catalytic properties. Adv Mater.

