# Supplementary material for: Highly Efficient Catalysis of Azo Dyes Using Recyclable Silver Nanoparticles Immobilized on Tannic Acid-Grafted Eggshell Membrane
Source: Nanoscale Res Lett. 2016 Oct 1;11:440. doi: 10.1186/s11671-016-1647-7 (PMC5052158; doi:10.1186/s11671-016-1647-7)
Supplement: Additional file 1: Figure S1. — The catalytic effects of Tan-ESM and AgNPs@Tannin-ESM composites on the reduction of CR (488 nm) and MO (446 nm). Figure S2. The time-dependent UV-vis absorption spectra of Congo red (a, c) and methyl orange (b, d) using AgNPs@Tan-ESM and NaBH4 separately. (DOCX 194 kb) [file 11671_2016_1647_MOESM1_ESM.docx]

Supporting Information for

Efficient Catalysis of Azo Dyes Using Recyclable Silver Nanoparticles Immobilized on Tannic Acid Grafted Eggshell Membrane

Xiaojing Liu ^a^, Miao Liang ^a^, Mingyue Liu ^a^, Rongxin Su ^a,b,c*^, Mengfan Wang ^a,c*^,

Wei Qi ^a,b,c^, Zhimin He ^a^

*^a^ State Key Laboratory of Chemical Engineering, School of Chemical Engineering and Technology, Tianjin University, Tianjin 300072, P. R. China.*

*^b^ Collaborative Innovation Center of Chemical Science and Engineering (Tianjin), Tianjin 300072, P. R. China.*

*^c^ Tianjin Key Laboratory of Membrane Science and Desalination Technology, Tianjin, 300072, China*





**Fig. S1.** The catalytic effects of Tan-ESM and AgNPs@Tannin-ESM composites on the reduction of CR (488 nm) and MO (446 nm).

**
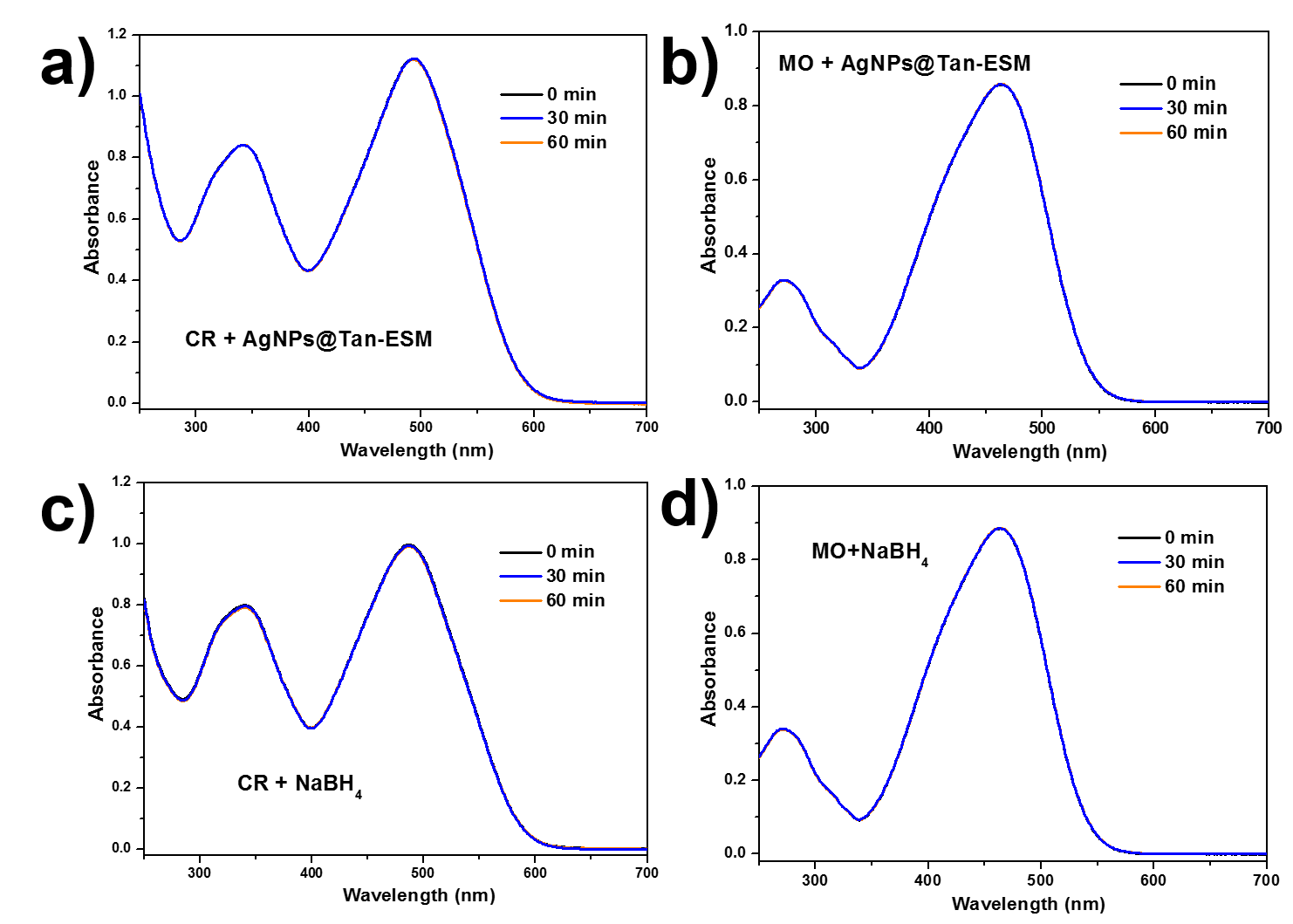
**

**Fig. S2.** The time-dependent UV-vis absorption spectra of Congo red (a, c) and methyl orange (b, d) using AgNPs@Tan-ESM and NaBH_4_ separately.
